# Hypertensive Disorders of Pregnancy and Pre-Pregnancy Hypertension with Subsequent Incident Venous Thromboembolic Events

**DOI:** 10.3390/ijerph21010089

**Published:** 2024-01-12

**Authors:** Angela M. Malek, Dulaney A. Wilson, Tanya N. Turan, Julio Mateus, Daniel T. Lackland, Kelly J. Hunt

**Affiliations:** 1 Department of Public Health Sciences, Medical University of South Carolina, Charleston, SC 29425, USA; 2 Department of Neurology, Medical University of South Carolina, Charleston, SC 29425, USA; 3 Atrium Health, Department of Obstetrics & Gynecology, Maternal-Fetal Medicine Division, Charlotte, NC 28204, USA

**Keywords:** hypertensive disorders of pregnancy, pre-pregnancy hypertension, maternal outcomes, venous thromboembolism, pulmonary embolism, deep vein thrombosis, race and ethnicity

## Abstract

Hypertensive disorders of pregnancy (HDP) and pre-pregnancy hypertension contribute to maternal morbidity and mortality. We examined the association of HDP and pre-pregnancy hypertension with subsequent venous thromboembolic (VTE) events. The retrospective cohort study included 444,859 women with ≥1 live, singleton birth in South Carolina (2004–2016). Hospital and emergency department visit and death certificate data defined incident VTE, HDP, and pre-pregnancy hypertension. Birth certificate data also defined the exposures. Adjusted Cox proportional hazards methods modeled VTE events risk. Of the cohort, 2.6% of women had pre-pregnancy hypertension, 5.8% had HDP, 2.8% had both pre-pregnancy hypertension and HDP (both conditions), and 88.8% had neither condition. The risk of incident VTE events within one year of delivery was higher in women with HDP (hazard ratio [HR] = 1.62, 95% confidence interval [CI]: 1.15–2.29) and both conditions (HR = 2.32, 95% CI: 1.60–3.35) compared to those with neither condition as was the risk within five years for women with HDP (HR = 1.35, 95% CI: 1.13–1.60) and for women with both conditions (HR = 1.82, 95% CI: 1.50–2.20). One- and five-year risks did not differ in women with pre-pregnancy hypertension compared to women with neither condition. Compared to non-Hispanic White (NHW) women with neither condition, the incident VTE event risk was elevated within five years of delivery for NHW (HR = 1.29, 95% CI: 1.02–1.63; HR = 1.59, 95% CI: 1.16–2.17) and non-Hispanic Black (NHB; HR = 1.51, 95% CI: 1.16–2.96; HR = 2.08, 95% CI: 1.62–2.66) women with HDP and with both conditions, respectively, and for NHB women with pre-pregnancy hypertension (HR = 1.50, 95% CI: 1.09–2.07). VTE event risk was highest in women with HDP, and the event rates were higher in NHB women than in NHW women in the same exposure group.

## 1. Introduction

The incidence of venous thromboembolism (VTE) during pregnancy in the United States (U.S.) is low at up to 1.72 per 1000 deliveries [1]. During pregnancy, VTE risk is approximately five times higher, and in the 3 months subsequent to delivery is 60 times higher than in non-pregnant women [2]. This is of public health importance as VTE represents a leading cause of severe maternal morbidity and mortality [3,4]. Furthermore, VTE rates are higher among Black women compared to women of other races [1,2,3]. Hypertensive disorders of pregnancy (HDP) including gestational hypertension, preeclampsia and eclampsia are significant complications affecting nearly 10% of pregnancies [5,6]. Compared with non-Hispanic White (NHW) women, chronic hypertension and HDP rates are higher in non-Hispanic Black (NHB) women [7,8,9,10]. Moreover, the risk of maternal and fetal/neonatal serious morbidity and mortality from HDP is elevated and varies by race and ethnicity [10,11,12]. Post-pregnancy cardiovascular outcomes associated with HDP, including mortality, can occur long term [13,14,15,16].

Although the causes and treatments differ for VTE and arterial cardiovascular disease, the pathophysiology between them is shared to some extent [17,18,19,20,21]. Preeclampsia, a type of HDP, and thrombophilia share pathogenic mechanisms resulting in an increased risk of developing VTE. During pregnancy, the risk of VTE is higher, due to anatomic and physiologic changes including the occurrence of a hypercoagulable state of the blood [22]. VTE history is associated with an increased risk of preeclampsia and placenta-mediated pregnancy complications [23]. Thus, a biologically plausible mechanism for placenta-mediated complications including preeclampsia [24,25,26,27,28] and VTE may be thrombophilia or thrombosis [29,30,31]. While relationships have been reported between preeclampsia and other HDP with VTE during pregnancy or postpartum by international studies [32,33,34,35], U.S. studies and evidence regarding potential differences by race/ethnicity are lacking. Therefore, we aimed to evaluate the association between HDP and pre-pregnancy hypertension with incident VTE events among women within one and five years after delivery and over the entire study period (up to fourteen years). We examined the risk of post-pregnancy incident VTE events and related mortality, as well as racial and ethnic group differences.

## 2. Materials and Methods

The data used for this study cannot be shared due to the policies of the South Carolina (SC) Revenue and Fiscal Affairs (RFA) Office Health and Demographics Section, and the SC Department of Health and Environmental Control (DHEC). The policies of these data sources also require that small numbers, less than five, be reported as <5.

### 2.1. Study Design

For this retrospective cohort study, existing statewide administrative billing data for SC hospital discharge records and emergency department (ED) visit encounters from the SC RFA Office were utilized. We also used birth certificate and death certificate data available from SC DHEC. Institutional review board approval was received with the requirement for consent waived.

### 2.2. Study Population

Birth certificate data identified live births in SC hospitals. Information on the mothers’ demographics, diagnosis and procedure codes (International Classification of Diseases, Ninth and Tenth Revision, Clinical Modification [ICD-9-CM/ICD-10-CM], Supplementary Materials Table S1), patient dispositions, and lengths of stay were available from hospitalization/ED visit data. Death certificate data included demographics, payers, current/previous pregnancies, and causes of maternal death (primary, underlying, comorbid) information. The majority (97.5%) of birth certificates were successfully linked to maternal hospitalization/ED visit data by the SC RFA, with a unique identifier.

### 2.3. Inclusion and Exclusion Criteria

**Figure 1:**
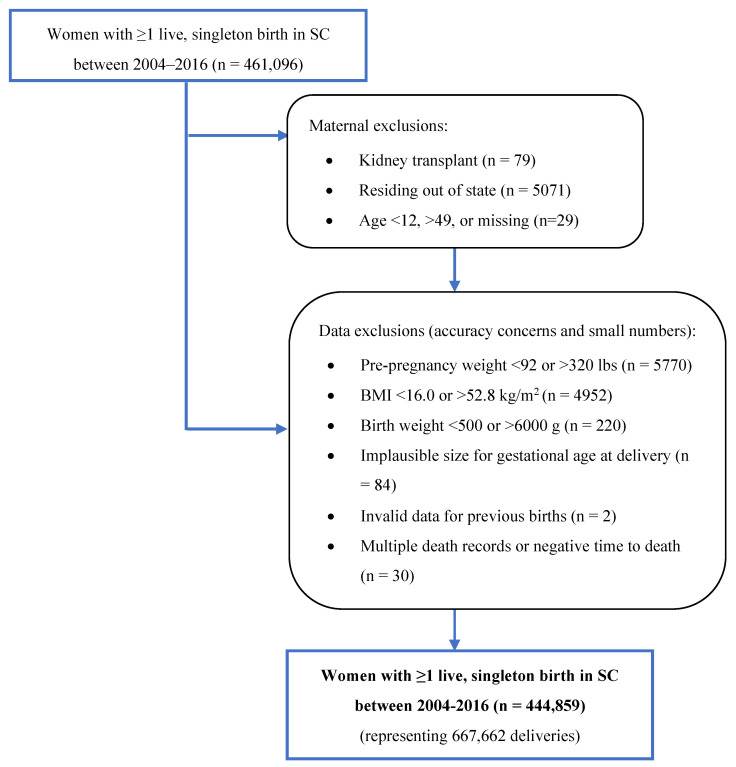
Study inclusion and exclusion criteria flowchart.

Our study included women with hospitalization/ED visit data and matching birth certificate data for single, liveborn infants delivered in SC hospitals between 2004 and 2016. Maternal hospitalization/ED visit and mortality data were obtained through 2017, providing one to fourteen years of follow-up. Figure 1 shows the study inclusion and exclusion criteria. We excluded out-of-state mothers who may not seek care regularly in SC and women with prior kidney transplants who may not be representative of the pre-pregnancy hypertension and HDP exposure groups. Because of small numbers and data accuracy concerns, a pre-pregnancy weight of <92 or >320 lbs, a pre-pregnancy BMI of <16.0 or >52.8 kg/m^2^, newborns with birth weights of <500 or >6000 g, and deliveries with implausible size for gestational age were excluded [36].

### 2.4. Definitions

Maternal information available from the birth certificate included self-reported race and ethnicity, education, primary payer, BMI at delivery and pre-pregnancy, smoking during and pre-pregnancy, pre-pregnancy and gestational hypertension, and pre-pregnancy and gestational diabetes mellitus. Self-reported race and ethnicity was categorized as NHW, NHB, Hispanic, or other race and ethnicity. Other race and ethnicity included Asian, American Indian, and women of other races and ethnicities. Prenatal care adequacy and utilization information based on the Revised-Graduated Prenatal Care Utilization Index (R-GINDEX) was available that combined the number of visits with the trimester and gestational age prenatal care began [37,38]. The six R-GINDEX categories are inadequate, intermediate, adequate, intensive, no care, or not able to calculate due to missing information, although quality of service is not evaluated.

Hospitalization/ED visit data included ICD-9/10-CM diagnosis and procedure codes used to define the exposure, outcome, and covariates (Table S1). The four exposure groups were women (1) with superimposed preeclampsia termed pre-pregnancy hypertension with superimposed HDP (referred to as “both conditions”), (2) with HDP without pre-pregnancy hypertension (“HDP”), (3) with pre-pregnancy hypertension without superimposed HDP (“pre-pregnancy HTN”), and (4) without pre-pregnancy hypertension or HDP (“neither condition”). HDP was defined by a birth certificate checkbox for gestational hypertension, which also included preeclampsia and eclampsia, or by hospitalization/ED visit data as gestational hypertension, preeclampsia, or eclampsia (ICD-9/10-CM: 642.3–642.7; O11–O16). Pre-pregnancy hypertension was defined by a birth certificate checkbox or hospitalization/ED visit data (ICD-9/10-CM: 642.0–642.2; O10). Pre-pregnancy hypertension with superimposed HDP was defined by birth certificate data and/or from hospitalization/ED visit data (ICD-9/10-CM: 642.7; O11–O12, O14–O15), with the understanding that this refers to preeclampsia. The first pregnancy in the dataset among women with neither condition and with HDP and/or pre-pregnancy hypertension was considered the index pregnancy. Diagnoses with ICD-9/10-CM codes in the hospitalization/ED visit and death certificate data as shown in Table S1 defined fatal and nonfatal maternal VTE events (VTE, PE, DVT) and arterial embolism or thrombosis. If an individual had multiple VTE events, only the first was counted. Cerebral venous thrombosis was also examined (ICD-9/10-CM codes: 437.6 and I67.6) although less than five events occurred throughout the study period.

### 2.5. Statistical Analysis

We used Chi-square tests to analyze categorical variables and analysis of variance to analyze continuous variables by group differences. Cox proportional hazards models were used to calculate HRs and corresponding 95% CIs to estimate incident VTE events/deaths within one and five years after delivery and over the entire study period (up to fourteen years after delivery). Schoenfeld residuals were used to check the proportional hazards assumption. Models included time from delivery to event or death and were adjusted for significant sociodemographic (maternal age at delivery; race and ethnicity; education; urban/rural residence; median household income per year; payer during pregnancy; Women, Infants, and Children [WIC] eligibility during pregnancy), behavioral (smoking during pregnancy), and clinical characteristics (pre-pregnancy BMI, change in BMI after delivery, pre-pregnancy or gestational diabetes mellitus, gestational age at delivery, mode of delivery [cesarean section (C-section) or vaginal], induced labor, number of pregnancies prior to index pregnancy, previous C-section, previous pre-term delivery, R-GINDEX). The R-GINDEX included a missing category, and previous C-section included an unknown category since this was one of the categories provided on the birth certificate. Sensitivity analyses were performed to examine the hazard of incident VTE events excluding arterial embolism within one and five years after delivery and over the entire study period (up to fourteen years after delivery).

For comparisons by racial and ethnic group, NHW women with neither condition served as the reference group and a joint analysis was carried out as VTE event rates varied at baseline. Hispanic and women of other racial/ethnic groups were included in the overall models but were excluded from race/ethnicity-specific models because of small numbers of incident VTE events especially when examined by exposure group (n = 96 and n = 25 throughout the study period, respectively). In a sensitivity analysis, the hazard of incident VTE events/deaths within one and five years of delivery as well as the entire study period (up to fourteen years after delivery) was examined among women stratified by race/ethnic group. Statistical analyses were performed using SAS Version 9.4 (SAS Institute Inc., Cary, NC, USA) [39]. Stata Version 13 was used to create graphs and to check the proportional hazards assumption [40].

## 3. Results

Of the 461,096 women with liveborn, singleton births in SC (2004–2016), 444,859 were eligible for the study including NHW (57.6%), NHB (30.9%), Hispanic (9.1%), and women of other races/ethnicities (2.4%). Pre-pregnancy hypertension without superimposed HDP (pre-pregnancy hypertension; 2.6%), no pre-pregnancy hypertension with HDP (HDP; 5.8%), and pre-pregnancy hypertension with superimposed HDP (both conditions; 2.8%) occurred in at least one pregnancy. The control group consisted of women with neither condition (88.8%) in pregnancies throughout the study period.

Sociodemographic and clinical characteristics are shown by exposure group in Table 1. Women with pre-pregnancy hypertension or both conditions were more likely than those with HDP or neither condition to be older and have a rural residence and a previous C-section. Women with pre-pregnancy hypertension, HDP, or both conditions were more likely to be NHB, WIC eligible during pregnancy, and to have pre-pregnancy or gestational diabetes mellitus, induced labor, Medicaid, a lower annual household income, a C-section, and a higher BMI pre-pregnancy than women with neither condition. They were also more likely to have a lower gestational age at delivery and to have received intensive or adequate (women with pre-pregnancy hypertension and HDP) prenatal care, as measured using the R-GINDEX than women with neither condition.

### 3.1. Incident VTE Events Subsequent to HDP and/or Pre-Pregnancy Hypertension

Table 2 displays the adjusted hazard of incident VTE events within one, five, and fourteen years of delivery by exposure status compared to women with neither condition. Within fourteen years of delivery, 3762 VTE events (n = 2356 PE, n = 1245 venous embolism, n = 121 arterial embolism) occurred; this also includes less than five cerebral venous thrombosis events and events not specified. Risk of VTE events within one year of delivery increased 62% (95% CI: 1.15–2.29) among women with HDP and 2.32-fold among women with both conditions (95% CI: 1.60–3.35) compared to women with neither condition. Risk of VTE events within five years of delivery was also elevated among women with HDP (HR = 1.35, 95% CI: 1.13–1.60) and both conditions (HR = 1.82, 95% CI: 1.50–2.20). Over the entire study period of up to fourteen years after delivery, risk of VTE events was increased for women with pre-pregnancy hypertension (HR = 1.36, 95% CI: 1.18–1.58), HDP (HR = 1.31, 95% CI: 1.16–1.48), and both conditions (HR = 1.74, 95% CI: 1.52–2.00) compared to women with neither condition.

A sensitivity analysis was performed in which the VTE definition was limited to venous VTE events (excluding arterial embolism). The adjusted hazards within one, five, and fourteen years of delivery by exposure status compared to women with neither condition are given in Table S2, Supplementary Materials; the results were not significantly different from those above.

### 3.2. Racial and Ethnic Differences in Incident VTE Events Following HDP and/or Pre-Pregnancy Hypertension

Racial and ethnic differences in incident VTE event rates and risk within five years of delivery are displayed in Table 3 for NHW and NHB women only, owing to a small number of events among Hispanic women and women of other race/ethnic groups. In general, NHB women had higher incident VTE event rates than their NHW counterparts regardless of the exposure. The number of incident VTE events was small in women with pre-pregnancy hypertension. The VTE event rate (per 1000 person-years) within one year in women with pre-pregnancy hypertension was 0.97 in NHW women and 0.51 in NHB women; within five years, the rates were 0.89 and 1.48, respectively; and incident VTE rates for the full period of follow-up were 1.48 in NHW women and 2.33 in NHB women.

**Figure 2:**
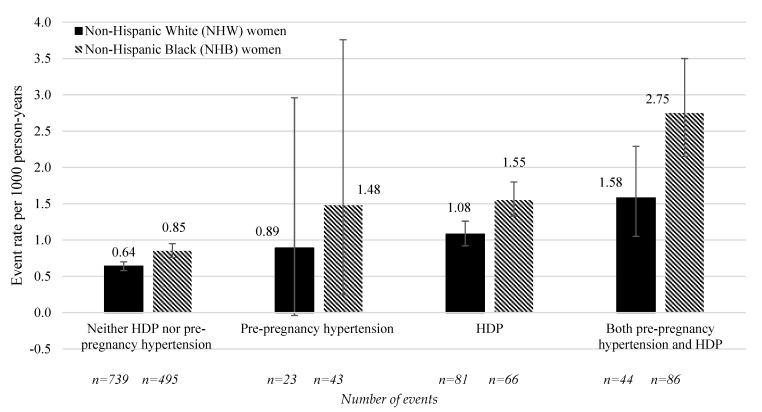
Maternal incident venous thromboembolic (VTE) events (per 1000 person-years) up to five years after delivery by exposure and racial and ethnic group. HDP, hypertensive disorders of pregnancy.

The VTE event rate (per 1000 person-years) within one, five, and fourteen years in women with HDP was higher in NHB women (1.99, 1.55, and 2.11) than in NHW women (1.46, 1.08, and 1.33), respectively. Similarly, the VTE event rate (per 1000 person-years) within one, five and fourteen years in women with both conditions was higher in NHB women (4.29, 2.75, and 3.07) than in NHW women (2.14, 1.58, and 2.12), respectively. Figure 2 presents race-specific incident VTE event rates per 1000 person-years (95% CI) for NHW women and NWB women within five years of delivery.

The adjusted hazard of maternal incident VTE events was compared among NHW and NHB women in a joint analysis with NHW women with neither condition serving as the reference group (Table 3). NHB women with both conditions experienced an increased risk of incident VTE events within one (HR = 2.90, 95% CI: 1.80–4.66) and five years of delivery (HR = 2.08, 95% CI: 1.62–2.66) as well as over the entire study period (up to fourteen years after delivery; HR = 2.13, 95% CI: 1.78–2.55) compared to NHW women with neither condition. Compared to NHW women with neither condition, NHW women with both conditions had an increased risk of incident VTE events within one (HR = 2.01, 95% CI: 1.10–3.68) and five (HR = 1.59, 95% CI: 1.16–2.17) years of delivery and over the entire study period (HR = 1.79, 95% CI: 1.43–2.23).

Among women with HDP, the incident VTE event risk was elevated for NHW and NHB women within one (HR = 1.64, 95% CI: 1.02–2.63 and HR = 1.87, 95% CI: 1.80–4.66), five years of delivery (HR = 1.29, 95% CI: 1.02–1.63 and HR = 1.51, 95% CI: 1.16–1.96), and over the entire study period (HR = 1.29, 95% CI: 1.09–1.54 and HR = 1.69, 95% CI: 1.41–2.02), respectively, compared to NHW women with neither condition.

Women with pre-pregnancy hypertension had a small number of incident VTE events. NHW women with pre-pregnancy hypertension did not differ from NHW women with neither condition within one or five years after delivery but did have a significantly increased risk of VTE over the entire study period (HR = 1.32, 95% CI: 1.03–1.69). NHB women with pre-pregnancy hypertension did not differ from NHW women with neither condition within one year of delivery but did show a significantly increased risk of VTE within five years after delivery (HR = 1.50, 95% CI: 1.09–2.07) and over the entire study period (HR = 1.78, 95% CI: 1.48–2.15).

Compared to NHW women with neither condition, NHB women with neither condition had an increased risk of incident VTE events over the entire study period (HR = 1.27, 95% CI: 1.17–1.38). An interaction between the exposure and racial and ethnic groups with incident VTE events was tested for but not significant (*p* = 0.72 within one, *p* = 0.46 within five, and *p* = 0.95 within fourteen years of delivery [study period]). Table S3, Supplementary Materials, presents event rates and the adjusted hazard of incident VTE events within one and five years of delivery and the entire study period (up to fourteen years after delivery) among women stratified by race/ethnic group. In general, the HRs were similar to those of the racial/ethnic-specific joint models or attenuated, remaining significant except for NHB women with HDP compared to NHB women with neither condition. However, the results of these models must be interpreted with caution, as the baseline rates of VTE events differ in NHB and NHW women.

## 4. Discussion

In a statewide retrospective cohort study in SC, we evaluated the relationship between HDP and pre-pregnancy hypertension with short-term (within one year) and long-term (within five years and throughout the study period of up to fourteen years after delivery) maternal post-pregnancy VTE events. Increased risk of incident VTE events was observed for women with HDP and/or pre-pregnancy hypertension with event rates highest for NHB women. Compared with NHW women, NHB women experienced higher risk of incident VTE events.

Our overall findings are consistent with those of recent studies. A Dutch nationwide cohort study (n = 1,919,918; 24,531,118 person-years) reported the adjusted risk of VTE during pregnancy and ≤3 months after delivery was doubled in women with hypertension during pregnancy compared to those with pregnancy complications and was nearly 8-fold higher among women with preeclampsia than women without pregnancy complications [35]. Over the follow-up period (≤13 years after first delivery), women with hypertension during pregnancy (1.5-fold) and preeclampsia (2.1-fold) were at increased VTE risk after adjustment [35]. Moreover, the risk attenuated slightly although it remained significant over the follow-up period after limiting VTE events to >3 months postpartum [35]. The aforementioned study did not consider pre-pregnancy hypertension or involve deaths. We were interested in including women exposed to HDP as well as pre-pregnancy hypertension to evaluate fatal and non-fatal incident VTE events across four exposure groups compared to women with neither condition. Thus, our findings provide an important contribution to the literature and evidence from a U.S. study as many studies have been international.

Most recently, in a nationwide cohort study in Denmark with 10.2 years median follow-up, a 43% elevated risk of VTE was observed among primiparous women with and without preeclampsia following adjustment [31]. A 2020 review by Hart et al. reported a number of pregnancy-related factors that have been associated with VTE including preeclampsia (3-fold increase) [22]. The odds of VTE were 1.5 times higher among women with prior HDP than without prior HDP after adjustment for age at enrollment and race in the prospective, observational United Kingdom Biobank study [41]. Studies conducted in Norway (registry-based case-control) and Canada (retrospective cohort) found women with preeclampsia compared to those without preeclampsia had a 3.8-fold increase in odds [32] and a 2.2-fold higher risk [33] of post-pregnancy VTE after covariate adjustment, respectively. Women with eclampsia experienced a 4.4-fold increase in the odds of post-pregnancy VTE than those without eclampsia after adjustment in the Norwegian study [32]. Increased postpartum VTE event risk was similarly reported to be tripled among women with preeclampsia after adjustment by a Swedish retrospective population-based study [34]. Using the U.S. Healthcare Cost and Utilization Project National Readmissions Database, of the more than 6 million delivery hospitalizations between 2013 and 2014, VTE readmissions within 60 days of discharge were estimated as being 4.7/10,000 [42]. Preeclampsia and gestational hypertension diagnoses were present among 8.2% and 19.7% of women with VTE readmissions, respectively (*p* < 0.01) [42]. Women with preeclampsia or gestational hypertension had twice the odds of being readmitted for VTE [42]. Potential race or ethnic differences could not be examined as this information was not available. However, not all studies have found a relationship. The U.S. National Inpatient Sample (2000–2001) did not observe an independent association between preeclampsia and gestational hypertension with VTE (OR = 0.9, 95% CI: 0.7–1.0), although certain medical conditions including hypertension increased the risk of pregnancy-related VTE 1.8-fold [43].

We had the advantage of being able to compare racial/ethnic differences among NHW and NHB women, whereas other studies were limited in their ability to assess potential racial or ethnic differences due to homogeneous populations [32,33,34] with only one study assessing descent (Dutch, Hindustani, other) in VTE risk among women with hypertension during pregnancy [35]. NHB women are a critical group to study because of their high risk of cardiovascular disease risk factors as well as HDP and related outcomes in general [44,45]. In a U.S. statewide case-control study, the odds of VTE at delivery or during the three months postpartum were 1.5-fold higher for Black women and 0.67-fold lower for Asian women than NHW women after covariate adjustment including preeclampsia [46]. Among White and Black women with adverse pregnancy outcomes, any thrombophilia was found to be more common among White women due primarily to Factor V Leiden mutation, whereas Black women were more likely to be diagnosed with protein S or antithrombin deficiencies [47]. A history of pregnancy loss(es), preeclampsia, preterm delivery, placental abruption, and intrauterine growth restriction defined adverse pregnancy outcomes with pregnancy losses making up about 90% [47]. Sickle cell disease or other comorbidities, resistance to fibrinolysis, and higher coagulation-factor levels (e.g., fibrinogen) are possible reasons that Black women are more likely to experience pregnancy-associated VTE [48,49]. However, the evidence is lacking, and a gap currently exists in the literature regarding differences that may exist by race/ethnicity in the relationship between HDP and VTE. Our study is the first to our knowledge to compare the risk of VTE following HDP by race/ethnicity. The public health implications of the findings from the current study as well as future investigations include the possibility of improved screening and monitoring of women of racial/ethnic minority groups and other subgroups found to be at high risk of adverse pregnancy-related outcomes. Additional research could be conducted to identify factors associated with health disparities in the relationship between HDP and VTE. This, in turn, could contribute to a reduction in poor pregnancy-associated outcomes for women with pre-existing risk factors including comorbidities, especially those at high risk.

VTE risk is higher in pregnancy, during which anatomic and physiologic changes are present including the occurrence of a hypercoagulable state of blood [22]. Physiologic anticoagulants are decreased, whereas some coagulation factors are increased in pregnancy. Preeclampsia begins with abnormal placentation followed by induced endothelial damage [50] that can possibly also result from delivery-related trauma. As the uterus expands, the inferior vena cava and pelvic veins in the lower extremities are compressed, and venous dilatation can result from progesterone levels [22]. Because the clinical characteristics are continuously changing, VTE is a dynamic condition [51] that can be due to hereditary and/or acquired causes. Hypercoagulability is also a cause of VTE in addition to damage to the vascular tissue and abnormal hemodynamics [52]. Hereditary risk factors for VTE include Factor V Leiden, Prothrombin G20210A, and deficiency in protein S, protein C, or antithrombin, whereas some therapies and many disorders (e.g., heart failure, obesity, pregnancy) are acquired risk factors [53]. High venous resistance, elevated coagulability and inflammation, as well as endothelial lining damage are related to higher VTE risk [52]. History of VTE has been reported to increase the risk of preeclampsia 50% as well as other placenta-mediated pregnancy complications [23]. A biologically plausible mechanism for placenta-mediated complications including preeclampsia [24,25,26,27,28] and VTE could be thrombophilia or thrombosis [29,30,31].

Women with a history of VTE events are treated with anticoagulation during pregnancy and postpartum. Anticoagulation is also used under other clinical circumstances including after C-section in patients with risk factors for VTE events such as obesity, admission with COVID-19-related complications, and prolonged hospitalization. For instance, all patients admitted to the antepartum unit for ≥72 h receive prophylactic anticoagulation. Although these preventive measures result in short-term VTE risk reduction, our findings are consistent with others that demonstrate the continued association of HDP with VTE events remote from delivery, independent of pre-pregnancy hypertension in the maternal venous system. It is also possible a shared biological risk factor increases the risk of both HDP and VTE [31].

### Strengths and Limitations

Our statewide population-based study included thirteen years of data on live, singleton births with up to fourteen years of follow-up available. Approximately 32% of the cohort included understudied NHB women, a group that experiences increased maternal morbidity and mortality. The administrative data sources used for this study provided a wealth of information. Prenatal care, delivery diagnoses (e.g., preterm delivery), information from previous births (e.g., C-section), self-reported race and ethnicity, parity, and other covariates (e.g., pre-pregnancy BMI and smoking) were available from birth certificate data. The R-GINDEX provided prenatal care information for approximately 72% of women with available data.

There are some limitations of this work. Our data sources may have underestimated the number of women in relation to the exposure given inconsistencies in the accuracy of preeclampsia and pre-pregnancy hypertension diagnosis codes [54]. To minimize the risk of information bias, all available data sources were used which included inpatient hospitalization discharge records, ED visit records, and birth certificates. Birth certificate and maternal hospitalization/ED visit data were able to be successfully linked for nearly all the cohort (97.5%). Using only hospitalization/ED visit and death certificate data, as we did not have access to outpatient data on incident VTE events, may have underestimated the outcome. Our data sources did not include information on onset, duration, severity, or HDP type such as preeclampsia, although models controlled for a proxy of HDP severity (continuous gestational age at delivery). Without access to medical records prior to delivery, our study was limited to pre-pregnancy hypertension as reported on the birth certificate which may result in underestimation. We also did not have information on previous history of VTE or clotting disorders or current use of blood thinners and thus were not able to exclude individuals with these conditions. There were a small number of events among women with pre-pregnancy hypertension resulting in low power. Few events were observed among Hispanic women and women of other races/ethnicities; therefore, racial and ethnic group differences could not be examined for these groups. Except for women who experienced hospitalization, an ED visit, or an event, we presumed loss to follow-up did not occur. While some women could have moved away from SC, our data sources could not determine this. Finally, we acknowledge the limitations of our retrospective observational study design which provides a weaker level of evidence compared to randomized and prospective studies.

## 5. Conclusions

In a retrospective cohort study, we investigated the association of HDP and pre-pregnancy hypertension with post-pregnancy VTE events up to one and five years after delivery as well as over the entire study period (within 14 years of delivery) in a diverse group of women who had one or more live, singleton births in SC. This study had the ability to compare differences by racial/ethnic group and to include a large number of cases and a long duration of follow-up, allowing examination of the outcome at multiple points. Our study reported an increased risk of VTE events among women with HDP regardless of the presence of pre-pregnancy hypertension. Compared with NHW women, NHB women in every exposure group and time period experienced higher rates of VTE events, except for pre-pregnancy hypertension within one year of delivery.

## Figures and Tables

**Table 1:** Index pregnancy characteristics of mothers who gave birth in South Carolina between 2004 and 2016 ^1,2,3^.

Characteristic		Total	Neither Pre-Pregnancy Hypertension nor HDP	Pre-Pregnancy Hypertension	HDP	Both Pre-Pregnancy Hypertension and HDP	
% or Mean ± SD		n = 444,859	n = 395,114 (88.8%)	n = 11,580 (2.6%)	n = 25,652 (5.8%)	n = 12,513 (2.8%)	*p*
Maternal age at delivery, y		27.2 ± 6.0	27.1 ± 6.0	28.9 ± 6.0	27.1 ± 6.2	30.3 ± 6.4	<0.0001
	<20	9.9	10.2	5.4	11.1	4.7	<0.0001
	20–24	26.4	26.9	20	26.6	16.4	
	25–29	28.4	28.6	28.3	28	23.5	
	30–34	22.3	22	27	21.1	27.5	
	35–39	10.6	10.1	15.2	10.4	20.7	
	40+	2.4	2.2	4.1	2.8	7.1	
Race and ethnicity							
	Non-Hispanic White	57.6	58.4	44.6	58.6	44.8	<0.0001
	Non-Hispanic Black	30.9	29.5	50.4	33.3	50.4	
	Hispanic	9.1	9.6	4.2	6.5	3.5	
	Other	2.4	2.5	0.8	1.6	1.2	
Education							
	Less than HS	18.2	18.6	17.4	16	12.7	<0.0001
	HS graduate	24.5	24.3	26.8	25.1	27.2	
	Some college	25.2	24.9	27.7	27.5	27.7	
	≥College graduate	32	32.1	28	31.5	32.3	
Rural residence		26.2	26.3	29.2	22.4	27	<0.0001
Annual household income							
	<$36,000	27	26.7	36.9	25.4	31.1	<0.0001
	$36,000 to <$54,000	47.2	47.1	43.9	49.5	47.9	
	≥$54,000	23.7	24	17.6	23.4	20.5	
Primary payer during pregnancy							
	Medicaid	48.2	47.9	55.3	49	50	<0.0001
	Private	39.8	39.7	37.8	41.5	41.7	
	Self-pay	5.3	5.6	3.3	3.3	2.4	
	Other	5.9	6	3	5.5	5	
WIC eligible in pregnancy		51.3	50.9	58	51.7	56.6	<0.0001
Smoking during pregnancy		11.7	11.8	12.3	10.9	11.3	<0.0001
Pre-pregnancy BMI (kg/m^2^)		27.2 ± 6.8	26.7 ± 6.4	32.3 ± 7.9	30.1 ± 7.6	33.4 ± 7.9	<0.0001
Gestational age at delivery		38.4 ± 1.9	38.6 ± 1.8	37.4 ± 2.6	37.4 ± 2.5	37.4 ± 2.6	<0.0001
Mode of delivery							
	Cesarean section	34.1	32.1	50.6	47.4	53.3	<0.0001
	Vaginal	65.9	67.9	49.3	52.6	46.7	
Induced labor		14.5	13.5	15.5	26.8	20.4	<0.0001
No. of pregnancies prior to index pregnancy		0.8 ± 1.1	0.8 ± 1.1	0.9 ± 1.2	0.6 ± 1.0	0.9 ± 1.2	<0.0001
Previous cesarean section							
	Yes	5.6	5.4	12.7	5.2	6.6	<0.0001
	No	41.1	40.8	36.5	47.8	42.1	
	Unknown	53.3	53.9	50.8	47	51.3	
Previous preterm birth		2.6	2.5	6.4	2.9	4.2	<0.0001
Prenatal care as measured via R-GINDEX							
	Inadequate	17.6	18.2	13.2	14.7	11.6	<0.0001
	Intermediate	22.5	23	18.8	20.5	16.5	
	Adequate	1.6	1.6	1.8	1.8	1.6	
	Intensive	28.8	27.5	39	36.4	44.7	
	No care	0.9	0.9	1	0.8	1	
	Missing	28.5	28.9	26.1	26	24.6	
Pre-pregnancy or gestational diabetes mellitus		6.7		16.3			<0.0001

BMI, body mass index; HDP, hypertensive disorders of pregnancy; HS, high school; no., number; R-GINDEX, Revised Graduated Prenatal Care Utilization Index; RUCA, rural–urban commuting area; SD, standard deviation; WIC, Women, Infants, and Children; y, years. ^1^ Data include person-level diagnostic codes at time of discharge or birth certificate. HDP were defined as pre-eclampsia, eclampsia, or gestational hypertension (ICD-9/10-CM: 642.3–642.7; O11–O16) based on hospitalization/emergency department (ED) visit data or gestational hypertension as reported on the birth certificate. Pre-pregnancy hypertension was based on hospitalization/ED visit data (ICD-9/10-CM: 642.0–642.2; O10) or birth certificates. Pre-pregnancy hypertension with superimposed HDP was based on hospitalization/ED visit data (ICD-9/10-CM: 642.7; O11–O12, O14–O15) or a combination of the above diagnosis codes for HDP and pre-pregnancy hypertension. ^2^ Smoking during pregnancy, pre-pregnancy BMI, BMI at delivery, gestational age at delivery, mode of delivery, induced labor, number of pregnancies prior to the index pregnancy, previous cesarean section, pre-pregnancy diabetes, and gestational diabetes were available from the birth certificate. Gestational diabetes mellitus was also defined using hospitalization/ED visit data based on ICD-9/10-CM codes. ^3^ Variables with >0.1% missing data included annual household income, n = 9297; prenatal care as measured via R-GINDEX, n = 126,955; primary payer, n = 3339; and WIC eligibility in pregnancy, n = 7070.

**Table 2:** The adjusted hazard of fatal and nonfatal incident venous thromboembolic (VTE) events among women by exposure group within one and five years of delivery as well as the entire study period (up to fourteen years after delivery).

	Incident VTE Events			
	Event	Event Rate (95% CI) ^1^	HR (95% CI) ^2,3^	
**Within 1 year of delivery**				
Neither pre-pregnancy HTN nor HDP	254	0.64 (0.57–0.73)		Referent
Pre-pregnancy HTN	8	0.69 (0.35–1.38)	0.77	(0.36–1.66)
HDP	41	1.60 (1.18–2.17)	**1.62**	**(1.15–2.29)**
Both pre-pregnancy HTN and HDP	41	3.28 (2.42–4.46)	**2.23**	**(1.60–3.35)**
**Within 5 years of delivery**				
Neither pre-pregnancy HTN nor HDP	1283	0.65 (0.62–0.69)		Referent
Pre-pregnancy HTN	66	1.14 (0.90–1.46)	1.21	(0.93–1.57)
HDP	152	1.19 (1.01–1.39)	**1.35**	**(1.13–1.60)**
Both pre-pregnancy HTN and HDP	134	2.16 (1.82–2.55)	**1.82**	**(1.50–2.20)**
**Within 14 years of delivery**				
Neither pre-pregnancy HTN nor HDP	3019	0.91 (0.88–0.94)		Referent
Pre-pregnancy HTN	202	1.85 (1.62–2.13)	**1.36**	**(1.18–1.58)**
HDP	297	1.53 (1.37–1.71)	**1.31**	**(1.16–1.48)**
Both pre-pregnancy HTN and HDP	244	2.60 (2.29–2.94)	**1.74**	**(1.52–2.00)**

CI, confidence interval; HDP, hypertensive disorders of pregnancy; HR, hazard ratio; HTN, hypertension. ^1^ Per 1000 person-years. ^2^ Adjusted for sociodemographic characteristics (maternal age; race/ethnicity; education; rural/urban residence; annual household income; primary payer during pregnancy; Women, Infants and Children [WIC] eligibility during pregnancy), a behavioral characteristic (smoking during pregnancy), and clinical characteristics (pre-pregnancy body mass index [BMI], number of pregnancies after index pregnancy, gestational age at delivery, mode of delivery, induced labor, previous cesarean section [C-section], previous pre-term delivery, Revised Graduated Prenatal Care Utilization Index [R-GINDEX], and pre-pregnancy or gestational diabetes). ^3^ **Bold** denotes statistical significance.

**Table 3:** The adjusted hazard of fatal and nonfatal incident venous thromboembolic (VTE) events within one and five years of delivery as well as the entire study period (up to fourteen years after delivery) among women by race/ethnic group.

	Non-Hispanic White Women			Non-Hispanic Black Women			
Incident VTE Events	Event	Event Rate (95% CI) ^1^	HR (95% CI) ^2^	Event	Event Rate (95% CI) ^1^	HR (95% CI) ^2,3^	*p* ^4^
**Within 1 year of delivery**							
Neither pre-pregnancy HTN nor HDP	136	0.59 (0.50–0.70)	Referent	111	0.95 (0.79–1.15)	**1.28 (0.97–1.69)**	0.72
Pre-pregnancy HTN	5	0.97 (0.40–2.33)	1.18 (0.43–3.21)	<5	0.51 (0.17–1.60)	0.67 (0.21–2.13)	
HDP	22	1.46 (0.96–2.22)	**1.64 (1.02–2.63)**	17	1.99 (1.24–3.21)	**1.87 (1.10–3.18)**	
Both pre-pregnancy HTN and HDP	12	2.14 (1.22–3.77)	**2.01 (1.10–3.68)**	27	4.29 (2.94–6.26)	**2.90 (1.80–4.66)**	
**Within 5 years of delivery**							
Neither pre-pregnancy HTN nor HDP	739	0.64 (0.60–0.69)	Referent	495	0.85 (0.78–0.93)	1.07 (0.94–1.21)	0.46
Pre-pregnancy HTN	23	0.89 (0.59–1.34)	1.00 (0.64–1.54)	43	1.48 (1.10–1.99)	**1.50 (1.09–2.07)**	
HDP	81	1.08 (0.87–1.34)	**1.29 (1.02–1.63)**	66	1.55 (1.22–1.98)	**1.51 (1.16–1.96)**	
Both pre-pregnancy HTN and HDP	44	1.58 (1.17–2.12)	**1.59 (1.16–2.17)**	86	2.75 (2.22–3.39)	**2.08 (1.62–2.66)**	
**Study period (within 14 years of delivery)**							
Neither pre-pregnancy HTN nor HDP	1603	0.84 (0.80–0.88)	Referent	1310	1.31 (1.24–1.38)	**1.27 (1.17–1.38)**	0.95
Pre-pregnancy HTN	70	1.48 (1.17–1.87)	**1.32 (1.03–1.69)**	131	2.33 (1.96–2.77)	**1.78 (1.48–2.15)**	
HDP	149	1.33 (1.13–1.56)	**1.29 (1.09–1.54)**	141	2.11 (1.79–2.49)	**1.69 (1.41–2.02)**	
Both pre-pregnancy HTN and HDP	88	2.12 (1.72–2.61)	**1.79 (1.43–2.23)**	149	3.07 (2.62–3.61)	**2.13 (1.78–2.55)**	

CI, confidence interval; HDP, hypertensive disorders of pregnancy; HR, hazard ratio; HTN, hypertension. ^1^ Per 1000 person-years. ^2^ Adjusted for sociodemographic characteristics (maternal age; education; rural/urban residence; annual household income; primary payer during pregnancy; Women, Infants and Children [WIC] during pregnancy), a behavioral characteristic (smoking during pregnancy), and clinical characteristics (pre-pregnancy body mass index [BMI], number of pregnancies after index pregnancy, gestational age at delivery, mode of delivery, induced labor, previous cesarean section, (C-section) previous pre-term delivery, Revised Graduated Prenatal Care Utilization Index [R-GINDEX], pre-pregnancy or gestational diabetes). ^3^ **Bold** denotes statistical significance. ^4^ *p*-value for interaction between HDP and pre-pregnancy hypertension and race–ethnic group.

## Data Availability

Restrictions apply to the availability of these data. The data used for this study cannot be shared due to policies of the South Carolina (SC) Revenue and Fiscal Affairs (RFA) Office, the Health and Demographics Section, and the SC Department of Health and Environmental Control (DHEC).

## Supplementary Materials

The following supporting information can be downloaded at: https://www.mdpi.com/article/10.3390/ijerph21010089/s1, Table S1: International Classification of Diseases, Ninth and Tenth Revision, Clinical Modification (ICD-9/10-CM) codes for hospitalization and emergency department visit encounters or as reported on birth or death certificates to define the outcome, exposure, and covariates of interest. Table S2. The hazard of fatal and nonfatal incident venous thromboembolic (VTE) events excluding arterial embolism among women by exposure group within one and five years of delivery as well as the entire study period (up to fourteen years after delivery). Table S3. The adjusted hazard of fatal and nonfatal incident venous thromboembolic (VTE) events within one and five years of delivery as well as the entire study period (up to fourteen years after delivery) among women stratified by race/ethnic group.

## References

[1] Heit J.A., Kobbervig C.E., James A.H., Petterson T.M., Bailey K.R., Melton L.J. (2005). Trends in the incidence of venous thromboembolism during pregnancy or postpartum: A 30-year population-based study. Ann. Intern. Med..

[2] Pomp E.R., Lenselink A.M., Rosendaal F.R., Doggen C.J. (2008). Pregnancy, the postpartum period and prothrombotic defects: Risk of venous thrombosis in the MEGA study. J. Thromb. Haemost..

[3] James A.H., Jamison M.G., Brancazio L.R., Myers E.R. (2006). Venous thromboembolism during pregnancy and the postpartum period: Incidence, risk factors, and mortality. Am. J. Obstet. Gynecol..

[4] Friedman A.M., Ananth C.V. (2016). Obstetrical venous thromboembolism: Epidemiology and strategies for prophylaxis. Semin. Perinatol..

[5] Breathett K., Muhlestein D., Foraker R., Gulati M. (2014). Differences in preeclampsia rates between African American and Caucasian women: Trends from the National Hospital Discharge Survey. J. Womens Health.

[6] Parikh N.I., Gonzalez J.M., Anderson C.A.M., Judd S.E., Rexrode K.M., Hlatky M.A., Gunderson E.P., Stuart J.J., Vaidya D., American Heart Association Council on Epidemiology and Prevention (2021). Adverse Pregnancy Outcomes and Cardiovascular Disease Risk: Unique Opportunities for Cardiovascular Disease Prevention in Women: A Scientific Statement From the American Heart Association. Circulation.

[7] Johnson J.D., Louis J.M. (2020). Does race or ethnicity play a role in the origin, pathophysiology, and outcomes of preeclampsia? An expert review of the literature. Am. J. Obstet. Gynecol..

[8] Grobman W.A., Parker C.B., Willinger M., Wing D.A., Silver R.M., Wapner R.J., Simhan H.N., Parry S., Mercer B.M., Haas D.M. (2018). Racial Disparities in Adverse Pregnancy Outcomes and Psychosocial Stress. Obstet. Gynecol..

[9] Ghosh G., Grewal J., Mannisto T., Mendola P., Chen Z., Xie Y., Laughon S.K. (2014). Racial/ethnic differences in pregnancy-related hypertensive disease in nulliparous women. Ethn. Dis..

[10] Miranda M.L., Swamy G.K., Edwards S., Maxson P., Gelfand A., James S. (2010). Disparities in maternal hypertension and pregnancy outcomes: Evidence from North Carolina, 1994–2003. Public Health Rep..

[11] Roberts J.M., Rich-Edwards J.W., McElrath T.F., Garmire L., Myatt L., Global Pregnancy Collaboration (2021). Subtypes of Preeclampsia: Recognition and Determining Clinical Usefulness. Hypertension.

[12] Petersen E.E., Davis N.L., Goodman D., Cox S., Syverson C., Seed K., Shapiro-Mendoza C., Callaghan W.M., Barfield W. (2019). Racial/Ethnic Disparities in Pregnancy-Related Deaths—United States, 2007–2016. MMWR Morb. Mortal. Wkly. Rep..

[13] Smith G.N., Pudwell J., Walker M., Wen S.W. (2012). Ten-year, thirty-year, and lifetime cardiovascular disease risk estimates following a pregnancy complicated by preeclampsia. J. Obstet. Gynaecol. Can..

[14] Bellamy L., Casas J.P., Hingorani A.D., Williams D.J. (2007). Pre-eclampsia and risk of cardiovascular disease and cancer in later life: Systematic review and meta-analysis. BMJ.

[15] Brown M.C., Best K.E., Pearce M.S., Waugh J., Robson S.C., Bell R. (2013). Cardiovascular disease risk in women with pre-eclampsia: Systematic review and meta-analysis. Eur. J. Epidemiol..

[16] Ahmed R., Dunford J., Mehran R., Robson S., Kunadian V. (2014). Pre-eclampsia and future cardiovascular risk among women: A review. J. Am. Coll. Cardiol..

[17] Prandoni P., Bilora F., Marchiori A., Bernardi E., Petrobelli F., Lensing A.W., Prins M.H., Girolami A. (2003). An association between atherosclerosis and venous thrombosis. N. Engl. J. Med..

[18] Sorensen H.T., Horvath-Puho E., Pedersen L., Baron J.A., Prandoni P. (2007). Venous thromboembolism and subsequent hospitalisation due to acute arterial cardiovascular events: A 20-year cohort study. Lancet.

[19] Klok F.A., Mos I.C., Broek L., Tamsma J.T., Rosendaal F.R., de Roos A., Huisman M.V. (2009). Risk of arterial cardiovascular events in patients after pulmonary embolism. Blood.

[20] Roach R.E., Lijfering W.M., Flinterman L.E., Rosendaal F.R., Cannegieter S.C. (2013). Increased risk of CVD after VT is determined by common etiologic factors. Blood.

[21] Lijfering W.M., Flinterman L.E., Vandenbroucke J.P., Rosendaal F.R., Cannegieter S.C. (2011). Relationship between venous and arterial thrombosis: A review of the literature from a causal perspective. Semin. Thromb. Hemost..

[22] Hart C., Bauersachs R., Scholz U., Zotz R., Bergmann F., Rott H., Linnemann B. (2020). Prevention of Venous Thromboembolism during Pregnancy and the Puerperium with a Special Focus on Women with Hereditary Thrombophilia or Prior VTE-Position Paper of the Working Group in Women’s Health of the Society of Thrombosis and Haemostasis (GTH). Hamostaseologie.

[23] Hansen A.T., Schmidt M., Horvath-Puho E., Pedersen L., Rothman K.J., Hvas A.M., Sorensen H.T. (2015). Preconception venous thromboembolism and placenta-mediated pregnancy complications. J. Thromb. Haemost..

[24] Dimitriadis E., Rolnik D.L., Zhou W., Estrada-Gutierrez G., Koga K., Francisco R.P.V., Whitehead C., Hyett J., da Silva Costa F., Nicolaides K. (2023). Pre-eclampsia. Nat. Rev. Dis. Primers.

[25] Mazzolai L., Ageno W., Alatri A., Bauersachs R., Becattini C., Brodmann M., Emmerich J., Konstantinides S., Meyer G., Middeldorp S. (2022). Second consensus document on diagnosis and management of acute deep vein thrombosis: Updated document elaborated by the ESC Working Group on aorta and peripheral vascular diseases and the ESC Working Group on pulmonary circulation and right ventricular function. Eur. J. Prev. Cardiol..

[26] Konstantinides S.V., Meyer G., Becattini C., Bueno H., Geersing G.J., Harjola V.P., Huisman M.V., Humbert M., Jennings C.S., Jimenez D. (2020). 2019 ESC Guidelines for the diagnosis and management of acute pulmonary embolism developed in collaboration with the European Respiratory Society (ERS). Eur. Heart J..

[27] Say L., Chou D., Gemmill A., Tuncalp O., Moller A.B., Daniels J., Gulmezoglu A.M., Temmerman M., Alkema L. (2014). Global causes of maternal death: A WHO systematic analysis. Lancet Glob. Health.

[28] Virkus R.A., Lokkegaard E., Lidegaard O., Langhoff-Roos J., Nielsen A.K., Rothman K.J., Bergholt T. (2014). Risk factors for venous thromboembolism in 1.3 million pregnancies: A nationwide prospective cohort. PLoS ONE.

[29] Rodger M.A., Langlois N.J., de Vries J.I., Rey E., Gris J.C., Martinelli I., Schleussner E., Ramsay T., Mallick R., Skidmore B. (2014). Low-molecular-weight heparin for prevention of placenta-mediated pregnancy complications: Protocol for a systematic review and individual patient data meta-analysis (AFFIRM). Syst. Rev..

[30] Stillbirth Collaborative Research Network Writing Group (2011). Causes of death among stillbirths. JAMA.

[31] Havers-Borgersen E., Butt J.H., Johansen M., Petersen O.B., Ekelund C.K., Rode L., Olesen J.B., Kober L., Fosbol E.L. (2023). Preeclampsia and Long-Term Risk of Venous Thromboembolism. JAMA Netw. Open.

[32] Jacobsen A.F., Skjeldestad F.E., Sandset P.M. (2008). Incidence and risk patterns of venous thromboembolism in pregnancy and puerperium—A register-based case-control study. Am. J. Obstet. Gynecol..

[33] Van Walraven C., Mamdani M., Cohn A., Katib Y., Walker M., Rodger M.A. (2003). Risk of subsequent thromboembolism for patients with pre-eclampsia. BMJ.

[34] Lindqvist P., Dahlback B., Marsal K. (1999). Thrombotic risk during pregnancy: A population study. Obstet. Gynecol..

[35] Scheres L.J.J., Lijfering W.M., Groenewegen N.F.M., Koole S., de Groot C.J.M., Middeldorp S., Cannegieter S.C. (2020). Hypertensive Complications of Pregnancy and Risk of Venous Thromboembolism. Hypertension.

[36] Alexander G.R., Himes J.H., Kaufman R.B., Mor J., Kogan M. (1996). A United States national reference for fetal growth. Obstet. Gynecol..

[37] Alexander G.R., Kotelchuck M. (1996). Quantifying the adequacy of prenatal care: A comparison of indices. Public. Health Rep..

[38] Tayebi T., Hamzehgardeshi Z., Ahmad Shirvani M., Dayhimi M., Danesh M. (2014). Relationship between Revised Graduated Index (R-GINDEX) of prenatal care utilization & preterm labor and low birth weight. Glob. J. Health Sci..

[39] SAS Institute (2012). Statistical Analytical Software.

[40] StataCorp (2013). Stata Statistical Software.

[41] Honigberg M.C., Zekavat S.M., Aragam K., Klarin D., Bhatt D.L., Scott N.S., Peloso G.M., Natarajan P. (2019). Long-Term Cardiovascular Risk in Women with Hypertension During Pregnancy. J. Am. Coll. Cardiol..

[42] Wen T., Wright J.D., Goffman D., D’Alton M.E., Mack W.J., Attenello F.J., Friedman A.M. (2018). Postpartum venous thromboembolism readmissions in the United States. Am. J. Obstet. Gynecol..

[43] Simpson E.L., Lawrenson R.A., Nightingale A.L., Farmer R.D. (2001). Venous thromboembolism in pregnancy and the puerperium: Incidence and additional risk factors from a London perinatal database. BJOG Int. J. Obstet. Gynaecol..

[44] Garovic V.D., Dechend R., Easterling T., Karumanchi S.A., McMurtry Baird S., Magee L.A., Rana S., Vermunt J.V., August P., American Heart Association Council on Hypertension (2022). Hypertension in Pregnancy: Diagnosis, Blood Pressure Goals, and Pharmacotherapy: A Scientific Statement from the American Heart Association. Hypertension.

[45] Virani S.S., Alonso A., Aparicio H.J., Benjamin E.J., Bittencourt M.S., Callaway C.W., Carson A.P., Chamberlain A.M., Cheng S., Delling F.N. (2021). Heart Disease and Stroke Statistics-2021 Update: A Report From the American Heart Association. Circulation.

[46] Blondon M., Harrington L.B., Righini M., Boehlen F., Bounameaux H., Smith N.L. (2014). Racial and ethnic differences in the risk of postpartum venous thromboembolism: A population-based, case-control study. J. Thromb. Haemost..

[47] Philipp C.S., Faiz A.S., Beckman M.G., Grant A., Bockenstedt P.L., Heit J.A., James A.H., Kulkarni R., Manco-Johnson M.J., Moll S. (2014). Differences in thrombotic risk factors in black and white women with adverse pregnancy outcome. Thromb. Res..

[48] Lutsey P.L., Wassel C.L., Cushman M., Sale M.M., Divers J., Folsom A.R. (2012). Genetic admixture is associated with plasma hemostatic factor levels in self-identified African Americans and Hispanics: The Multi-Ethnic Study of Atherosclerosis. J. Thromb. Haemost..

[49] Montagnana M., Favaloro E.J., Franchini M., Guidi G.C., Lippi G. (2010). The role of ethnicity, age and gender in venous thromboembolism. J. Thromb. Thrombolysis.

[50] Phipps E.A., Thadhani R., Benzing T., Karumanchi S.A. (2019). Pre-eclampsia: Pathogenesis, novel diagnostics and therapies. Nat. Rev. Nephrol..

[51] Kroegel C., Reissig A. (2003). Principle mechanisms underlying venous thromboembolism: Epidemiology, risk factors, pathophysiology and pathogenesis. Respiration.

[52] Watson C., Saaid H., Vedula V., Cardenas J.C., Henke P.K., Nicoud F., Xu X.Y., Hunt B.J., Manning K.B. (2023). Venous Thromboembolism: Review of Clinical Challenges, Biology, Assessment, Treatment, and Modeling. Ann. Biomed. Eng..

[53] Bauer K.A., Lip G.Y.H. Overview of the Causes of Venous Thrombosis. https://www.uptodate.com/contents/overview-of-the-causes-of-venous-thrombosis?search=vte%20risk%20factors&source=search_result&selectedTitle=1~150&usage_type=default&display_rank=1#H4.

[54] Geller S.E., Ahmed S., Brown M.L., Cox S.M., Rosenberg D., Kilpatrick S.J. (2004). International Classification of Diseases-9th revision coding for preeclampsia: How accurate is it?. Am. J. Obstet. Gynecol..

