# Supplementary material for: Hypertensive Disorders of Pregnancy and Pre-Pregnancy Hypertension with Subsequent Incident Venous Thromboembolic Events
Source: Int J Environ Res Public Health. 2024 Jan 12;21(1):89. doi: 10.3390/ijerph21010089 (PMC10815509; doi:10.3390/ijerph21010089)
Supplement: Supplementary file 1 [file ijerph-21-00089-s001.zip › ijerph-2707192-supplementary.pdf]

Table S1. International Classification of Diseases, Ninth and Tenth Revision, Clinical Modification (ICD-9/10-CM) codes for hospitalization and emergency department visit encounters or as reported on birth or death certificates to define the outcome, exposure, and covariates of interest

DIAGNOSIS	ICD-9-CM Code	ICD-10-CM Code
**Outcome of delivery, birth [1]**	V27, V30, 640-649, 650-659, 660-669	Z37.x
**Single liveborn, single live birth [2]**	V27.0, V30.0	Z37.0
**Diabetes (maternal/gestational)**	250.00-250.92, 648.0, 648.8	E10, E11, O24.3, O24.4, O24.9
Pre-pregnancy and gestational diabetes (birth certificate)	--	--
**Venous thromboembolic events**<br>Deep vein thrombosis (DVT), Arterial embolism or thrombosis, Venous thromboembolism (VTE), Pulmonary embolism (PE), Cerebral venous thrombosis	415.x, 437.6, 444, 445, 451.1, 451.2, 451.81, 452, 453.x, V125.1	I26.x, I67.6, I74, I75, I80.1, I80.2, I80.3, I81, I82.x,
**Hypertension; complicating pregnancy**	642.0-642.3, 642.9	O10
**Hypertensive disorders of pregnancy (HDP)**<br>Gestational hypertension, Pre-eclampsia, Eclampsia,	642.3-642.7	O11-O16
Gestational hypertension (birth certificate)	--	--
**Kidney transplant**	procedure code: 55.6, V42.0	Z94.0
**Pre-pregnancy hypertension**<br>Benign essential hypertension complicating pregnancy, childbirth, and the puerperium; Hypertension secondary to renal disease complicating pregnancy, childbirth, and the puerperium; Other pre-existing hypertension complicating pregnancy, childbirth, and the puerperium; Pre-existing essential hypertension complicating pregnancy, childbirth, and the puerperium, Pre-existing hypertensive heart disease complicating pregnancy, childbirth and the puerperium.	642.0-642.2	O10
Pre-pregnancy hypertension (birth certificate)	--	--
**Pre-pregnancy hypertension with superimposed HDP [2]**<br>Pre-eclampsia or eclampsia superimposed on pre-existing hypertension; Pre-existing hypertension with pre-eclampsia	642.7	O11, O12, O14, O15

[1] Obtained from the mother’s hospitalization/emergency department visit record.

[2] A combination of diagnosis codes for hypertensive disorder of pregnancy and pre-pregnancy hypertension was also used to defne pre-pregnany hypertension with superimposed HDP.

Table S2. The adjusted hazard of fatal and nonfatal incident venous thromboembolic (VTE) events excluding arterial embolism among women by exposure group within one and five years of delivery as well as the entire study period (up to fourteen years after delivery)

	Incident VTE Events				
	**Event**	**Event Rate (95% CI)** [1]		**HR (95% CI)** [2]	
**Within 1 year of delivery**					
Neither pre-pregnancy HTN nor HDP	247	**0.63**	**(0.55-0.71)**		Referent
Pre-pregnancy HTN	8	0.69	(0.35-1.38)	0.82	(0.38-1.76)
HDP	38	**1.48**	**(1.08-2.04)**	**1.63**	**(1.14-2.32)**
Both pre-pregnancy HTN and HDP	40	**3.20**	**(2.35-4.37)**	**2.42**	**(1.66-3.52)**
**Within 5 years of delivery**					
Neither pre-pregnancy HTN nor HDP	1,256	**0.64**	**(0.60-0.67)**		Referent
Pre-pregnancy HTN	62	1.07	(0.84-1.38)	1.16	(0.89-1.52)
HDP	148	1.16	(0.99-1.36)	**1.36**	**(1.14-1.62)**
Both pre-pregnancy HTN and HDP	129	**2.08**	**(1.75-2.47)**	**1.80**	**(1.48-2.19)**
**Within 14 years of delivery**					
Neither pre-pregnancy HTN nor HDP	2,964	**0.89**	**(0.86-0.93)**		Referent
Pre-pregnancy HTN	194	**1.78**	**(1.55-2.05)**	**1.34**	**(1.15-1.56)**
HDP	290	**1.49**	**(1.33-1.68)**	**1.31**	**(1.16-1.49)**
Both pre-pregnancy HTN and HDP	237	**2.52**	**(2.22-2.86)**	**1.74**	**(1.51-2.00)**

CI, confidence interval; HDP, hypertensive disorders of pregnancy; HR, hazard ratio; HTN, hypertension

[1] Per 1,000 person-years.

[2] Adjusted for sociodemographic characteristics (maternal age; race/ethnicity; education; rural/urban residence; annual household income; primary payer during pregnancy; Women, Infants and Children [WIC] eligibility during pregnancy), a behavioral characteristic (smoking during pregnancy), and clinical characteristics (pre-pregnancy body mass index [BMI], number of pregnancies after index pregnancy, gestational age at delivery, mode of delivery, induced labor, previous cesarean section [C-section], previous pre-term delivery, Revised-Graduated Prenatal Care Utilization Index [R-GINDEX], and pre-pregnancy or gestational diabetes).

Table S3. The adjusted hazard of fatal and nonfatal incident venous thromboembolic (VTE) events within one and five years of delivery as well as the entire study period (up to fourteen years after delivery) among women stratified by race/ethnic group

	**Non-Hispanic White Women**			**Non-Hispanic Black Women**			
**Incident VTE events**	Event	Event Rate (95% CI) [1]	HR (95% CI) [2]	Event	Event Rate (95% CI) [1]	HR (95% CI) [2]	***p*** [3]
**Within 1 year of delivery**							
Neither pre-pregnancy HTN nor HDP	136	**0.59 (0.50-0.70)**	Referent	111	0.95 (0.79-1.15)	Referent	0.72
Pre-pregnancy HTN	5	0.97 (0.40-2.33)	1.20 (0.43-3.29)	<5	0.51 (0.17-1.60)	0.52 (0.16-1.66)	
HDP	22	1.46 (0.96-2.22)	**1.69 (1.05-2.75)**	17	**1.99 (1.24-3.21)**	1.43 (0.84-2.42)	
Both pre-pregnancy HTN and HDP	12	**2.14 (1.22-3.77)**	**2.09 (1.13-3.88)**	27	**4.29 (2.94-6.26)**	**2.17 (1.34-3.51)**	
**Within 5 years of delivery**							
Neither pre-pregnancy HTN nor HDP	739	**0.64 (0.60-0.69)**	Referent	495	**0.85 (0.78-0.93)**	Referent	0.46
Pre-pregnancy HTN	23	0.89 (0.59-1.34)	0.95 (0.61-1.47)	43	**1.48 (1.10-1.99)**	**1.49 (1.08-2.07)**	
HDP	81	1.08 (0.87-1.34)	**1.28 (1.01-1.63)**	66	**1.55 (1.22-1.98)**	**1.42 (1.09-1.85)**	
Both pre-pregnancy HTN and HDP	44	**1.58 (1.17-2.12)**	**1.58 (1.15-2.17)**	86	**2.75 (2.22-3.39)**	**1.94 (1.51-2.49)**	
**Study period (within 14 years of delivery)**							
Neither pre-pregnancy HTN nor HDP	1,603	**0.84 (0.80-0.88)**	Referent	1,310	**1.31 (1.24-1.38)**	Referent	0.95
Pre-pregnancy HTN	70	**1.48 (1.17-1.87)**	1.25 (0.97-1.60)	131	**2.33 (1.96-2.77)**	**1.47 (1.22-1.78)**	
HDP	149	**1.33 (1.13-1.56)**	**1.24 (1.05-1.48)**	141	**2.11 (1.79-2.49)**	**1.38 (1.15-1.65)**	
Both pre-pregnancy HTN and HDP	88	**2.12 (1.72-2.61)**	**1.71 (1.37-2.14)**	149	**3.07 (2.62-3.61)**	**1.74 (1.45-2.09)**	

CI, confidence interval; HDP, hypertensive disorders of pregnancy; HR, hazard ratio; HTN, hypertension.

[1] Per 1,000 person-years.

[2]Adjusted for sociodemographic characteristics (maternal age; education; rural/urban residence; annual household income; primary payer during pregnancy; Women, Infants and Children [WIC] during pregnancy), a behavioral characteristic (smoking during pregnancy), and clinical characteristics (pre-pregnancy body mass index [BMI], number of pregnancies after index pregnancy, gestational age at delivery, mode of delivery, induced labor, previous cesarean section, (C-section) previous pre-term delivery, Revised-Graduated Prenatal Care Utilization Index [R-GINDEX], pre-pregnancy or gestational diabetes).

[3] p-value for interaction between HDP and pre-pregnancy hypertension and race-ethnic group.
